# Impact of Aortic Stenosis on Myofiber Stress: Translational Application of Left Ventricle-Aortic Coupling Simulation

**DOI:** 10.3389/fphys.2020.574211

**Published:** 2020-09-08

**Authors:** Andrew D. Wisneski, Yunjie Wang, Tobias Deuse, Arthur C. Hill, Salvatore Pasta, Kevin L. Sack, Jiang Yao, Julius M. Guccione

**Affiliations:** ^1^Department of Surgery, University of California, San Francisco, San Francisco, CA, United States; ^2^Thornton Tomassetti Lifesciences Division, Santa Clara, CA, United States; ^3^Department of Engineering, Universita degli Studi di Palermo, Palermo, Italy; ^4^Cardiovascular Research Division, Medtronic Inc., Minneapolis, MN, United States; ^5^Dassault Systèmes Simulia, Johnston, RI, United States

**Keywords:** aortic stenosis, finite element method, myofiber stress, ventricular function, realistic simulation, ventricle-aortic coupling

## Abstract

The severity of aortic stenosis (AS) has traditionally been graded by measuring hemodynamic parameters of transvalvular pressure gradient, ejection jet velocity, or estimating valve orifice area. Recent research has highlighted limitations of these criteria at effectively grading AS in presence of left ventricle (LV) dysfunction. We hypothesized that simulations coupling the aorta and LV could provide meaningful insight into myocardial biomechanical derangements that accompany AS. A realistic finite element model of the human heart with a coupled lumped-parameter circulatory system was used to simulate AS. Finite element analysis was performed with Abaqus FEA. An anisotropic hyperelastic model was assigned to LV passive properties, and a time-varying elastance function governed the LV active response. Global LV myofiber peak systolic stress (mean ± standard deviation) was 9.31 ± 10.33 kPa at baseline, 13.13 ± 10.29 kPa for moderate AS, and 16.18 ± 10.59 kPa for severe AS. Mean LV myofiber peak systolic strains were −22.40 ± 8.73%, −22.24 ± 8.91%, and −21.97 ± 9.18%, respectively. Stress was significantly elevated compared to baseline for moderate (*p* < 0.01) and severe AS (*p* < 0.001), and when compared to each other (*p* < 0.01). Ventricular regions that experienced the greatest systolic stress were (severe AS vs. baseline) basal inferior (39.87 vs. 30.02 kPa; *p* < 0.01), mid-anteroseptal (32.29 vs. 24.79 kPa; *p* < 0.001), and apex (27.99 vs. 23.52 kPa; *p* < 0.001). This data serves as a reference for future studies that will incorporate patient-specific ventricular geometries and material parameters, aiming to correlate LV biomechanics to AS severity.

## Introduction

Aortic stenosis (AS) is the most prevalent valvular heart disease in the developed world (Lindman et al., 2013; Go et al., 2014; Miura et al., 2015). Without treatment by surgical aortic valve replacement or transcatheter aortic valve replacement, AS leads to irreversible left ventricle (LV) remodeling and congestive heart failure, which have a poor prognosis (Lindman et al., 2013; Go et al., 2014; Miura et al., 2015). Traditional means of grading AS severity have predominantly relied on measuring hemodynamically derived parameters such as transvalvular pressure gradient, effective valve orifice area, and ejection blood jet velocity (Nishimura et al., 2014; Baumgartner et al., 2017). Recent research has brought to light limitations of these criteria at effectively grading AS in the presence of LV dysfunction coupled with decreased systemic arterial compliance, termed “low-flow, low-gradient” AS (Hachicha et al., 2007; Pibarot and Dumesnil, 2012; Tribouilloy et al., 2015). Additionally, the best way to manage severe AS in asymptomatic patients remains unclear, with only limited guiding evidence for the best course of treatment (Wachtell, 2008; Nishimura et al., 2014; Carter-Storch et al., 2019). Discordant outcomes have been reported on the role of aortic valve replacement for these groups of patients, highlighting the need to better diagnose and select appropriate patients for treatment (Hachicha et al., 2007; Pibarot and Dumesnil, 2012; Pibarot and Clavel, 2015; Tribouilloy et al., 2015; Chadha et al., 2019).

Rather than being viewed as an isolated valve disease, AS warrants a renewed understanding of its complex pathophysiology wherein its detrimental effects on the cardiovascular system are considered as derangements of the LV, the aortic valve, and systemic vasculature together (Briand et al., 2005). Advances in computational modeling techniques now enable AS to be studied from a cardiovascular systems perspective. LV-aortic coupling is a concept that describes the inter-dependency of the LV and the aorta/systemic blood vessels that impact cardiovascular function (Karabelas et al., 2018; Shavik et al., 2018; Ikonomidis et al., 2019). Multi-domain models of the human heart and circulatory system now offer a complete mechanistic model of the ventricles, aortic valve, and vasculature, making it ideally suited to investigate AS (Baillargeon et al., 2014; Genet et al., 2014, 2016; Dabiri et al., 2018; Sack et al., 2018b; Ghosh et al., 2020).

We believe that simulations of LV-aortic coupling with AS will enable us to identify LV biomechanical parameters that are prognostically significant markers of AS. Accordingly, as a first step, we created isolated AS in an idealized human heart model with the goal of gaining meaningful insight into the biomechanics of the actual end-organ, the LV, which AS treatment seeks to preserve. This investigation is one of the first of its kind to explore this critical aspect of AS pathophysiology with the aid of powerful computational simulation techniques.

## Methods

An idealized human heart model was generated from methods described in Baillargeon et al. (2014) comprising solid components, a finite element model, and a muscle fiber model. It represents an average heart in a middle-aged individual and can be altered to create diseased states.

### Solid Model of the Ventricle

**FIGURE 1:**
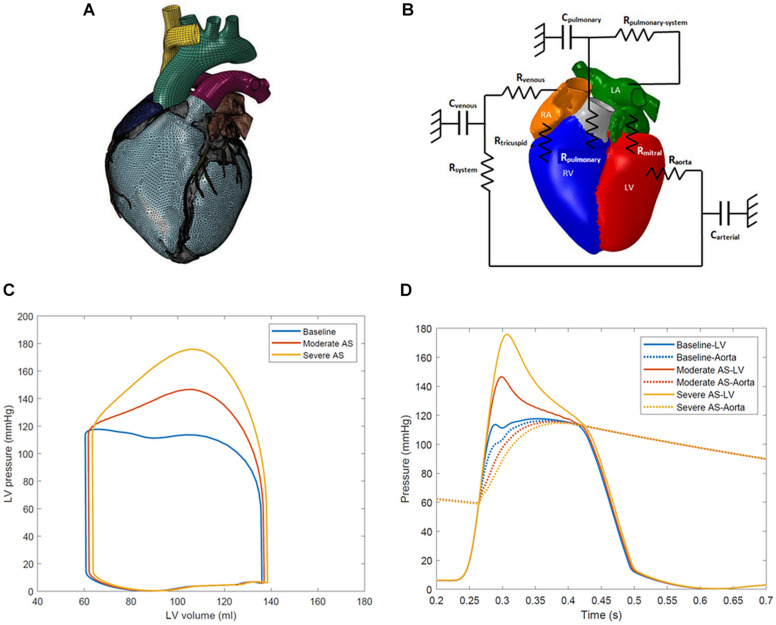
**(A)** Solid finite element model of the human heart model. **(B)** Schematic representation of lumped parameter model of circulatory system fluid links and parameters. The term R_aorta_ represents the aortic valve resistance parameter. R_mitral_, mitral valve resistance; C_arterial_, systemic arterial compliance; R_system_, systemic arterial resistance; C_venous_, venous compliance; R_venous_, systemic venous compliance; R_tricuspid_, tricuspid valve resistance; R_pulmonary_, pulmonary valve resistance; C_pulmonary_, pulmonary vascular compliance; R_pulmonary–system_, pulmonary vascular resistance. **(C)** Left ventricle pressure-volume loops for baseline, moderate aortic stenosis, and severe aortic stenosis conditions. **(D)** Left ventricle-aortic time pressure curves over one cardiac cycle. The systolic gradient between the left ventricle and aorta can be appreciated and serves as a marker of aortic stenosis severity.

The solid model of the human heart portrays realistic anatomy of four chambers, four valves, trabeculae in the ventricles, and great vessels including the ascending aorta, the aortic arch, pulmonary artery, and superior vena cava (Figure 1A). The LV finite element model comprises approximately 120,000 tetrahedral elements. Cardiac fiber orientation follows a rule-based approach from −60 degrees from epicardium to +60 degrees at the endocardium (Dabiri et al., 2018; Sack et al., 2018a). Fiber and sheet directions are interpolated and assigned to integration points of the finite element model (Wong and Kuhl, 2014).

### Constitutive Model Passive Material Description

The ventricular material model passive response uses the anisotropic hyperelastic formulation developed by Holzapfel and Ogden, and has been widely published in many cardiac modeling studies (Holzapfel and Ogden, 2009; Carrick et al., 2012; Sack et al., 2016, 2018a). The deviatoric response is governed by the following strain energy potential:

$$\Psi_{dev}=\frac{a}{2b}\text{exp}\left[b\left({\bar{I}}_{1}-3\right)\right]+\sum_{i=f,s}\frac{a_{i}}{2b_{i}}\left\{\text{exp}\left[b_{i}\left({\left({\bar{I}}_{4i}-1\right)}^{2}\right)\right]-1\right\}$$

(1) $$+\frac{a_{fs}}{2b_{fs}}[\text{exp}\left(b_{fs}{\bar{I}}_{8fs}^{2}-1\right]$$

Eight material parameters *a, b, a_*f*_, b_*f*,_ a_*s*_, b_*s*_, a_*fs*_, b_*fs*_*, and four strain invariants $\left({\bar{I}}_{1},{\bar{I}}_{4f},{\bar{I}}_{4s}{\bar{I}}_{8fs}^{2}\right)$define Equation (1). For these simulations, *a* = 3.354 kPa, *b* = 7.08, *a*_*f*_ = 2.501 kPa,*b_*f*_* = 5.34, while the remaining parameters were set to null. The strain invariants are derived from the isochoric right Cauchy-Green tensor:

(2) $$\bar{C}={\bar{F}}^{T}\bar{F}=J^{-2/3}C=J^{-2/3}F^{T}F$$

***F*** is the deformation gradient, *J* is the determinant of the deformation gradient, *J* = det (*F*) and $\bar{F}$ is the isochoric part of the deformation gradient where $\bar{F}=J^{-1/3}F$ and $\det\left(\bar{F}\right)=1$. The strain invariants can now be defined as:

(3) $${\bar{I}}_{1}=tr\left(\bar{C}\right),{\bar{I}}_{4f}=f_{0}\cdot \left(\bar{C}f_{0}\right),{\bar{I}}_{4s}=s_{0}\cdot \left(\bar{C}s_{0}\right),{\bar{I}}_{8fs}=f_{0}\cdot \left(\bar{C}s_{0}\right)$$

Terms ***f***_0_ and ***s***_0_ are orthogonal vectors in the fiber and sheet direction in the reference configuration. The volumetric response is governed by:

(4) $${\bar{\Psi }}_{vol}=\frac{1}{D}\left(\frac{\left(J^{2}-1\right)}{2}-\ln\left(J\right)\right)$$

where *J* is the third deformation gradient invariant, and *D* is the multiple of the bulk modulus $\left(D=\frac{2}{K}\right).$ K was set to 1000 kPa. This material model has been validated by Genet et al. for the purposes of ventricular computational modeling (Baillargeon et al., 2014; Genet et al., 2014).

### Active Material Description

The active myocardial tissue response is represented as a time-varying elastance model (Guccione and McCulloch, 1993; Walker et al., 2005):

(5) $$\sigma_{af}\left(t,E_{ff}\right)=\frac{T_{max}}{2}\frac{Ca_{0}^{2}}{Ca_{0}^{2}+ECa_{50}^{2}\left(E_{ff}\right)}\left(1-\text{cos}\left(\omega \left(t,E_{ff}\right)\right)\right)$$

with functions defined as:

(6) $$ECa_{50}\left(E_{ff}\right)=\frac{Ca_{0max}}{\sqrt{e^{B(l\left(E_{ff}\right)-l_{0}}-1}}$$

(7a) $$\omega \left(t,E_{ff}\right)=\pi \frac{t}{t_{0}}\;\text{when}0\leq t<t_{0}$$

$$\omega \left(t,E_{ff}\right)=\pi \frac{t-t_{0}+t_{r}\left(l\left(E_{ff}\right)\right)}{t_{r}}$$

(7b) $$\;\;\text{when}t_{0}\leq t\leq t_{0}+t_{r}\left(l\left(E_{ff}\right)\right)$$

(7c) $$\omega \left(t,E_{ff}\right)=0\;\text{when}t>t_{0}+t_{r}\left(l\left(E_{ff}\right)\right)$$

(7d) $$t_{r}\left(l\right)=ml+b$$

(7e) $$l\left(E_{ff}\right)=l_{r}\sqrt{2E_{ff}+1}$$

*T*_*max*_ is the maximum allowable active tension and is multiplied by terms regulating calcium concentration and the time course of the contraction. These two terms are dependent on the sarcomere length *l*. This law has been used extensively in prior published studies on ventricular mechanics (Walker et al., 2005; Carrick et al., 2012; Sack et al., 2016, 2018b). Parameters were set as follows: *T*_*max*_ = 135.7 kPa, *Ca*_0_ = 4.35 μmol/l, *Ca_0__*max*_* = 4.35 μmol/l, *m* = 1.0489 s μm^–1^, *b* = -1.429 s, *B* = 4.750 μm^–1^, *l*_0_ = 1.58 μm. *l*_*r*_ is the sarcomere length in the unloaded state, and was assumed to vary linearly from 1.78 μm at the endocardium to 1.91 μm at the epicardium (Guccione et al., 1993; Rodriguez et al., 1993; Walker et al., 2005).

The total stress (scalar form) in the sheet direction of the fiber is represented by:

(8) $$\sigma_{s}=\sigma_{ps}+n*\sigma_{af}$$

where active stress in the sheet direction, σ_*s*_, is the sum of passive stress, σ_*ps*_, and a portion of fiber direction stress, n*σ_*af*_. The parameter *n* is a scalar value less than 1.0 and represents the interaction between adjacent muscle fibers; a value of *n* = 0.4 was used (Walker et al., 2005).

### Circulatory System, Aortic Stenosis, and the Cardiac Cycle

The finite element model of the ventricles is coupled to lumped-parameter models of the pulmonary and systemic circulatory systems. This arrangement has been effective in other studies to link the ventricles and systemic circulation (Sack et al., 2016, 2018a, b). A schematic of the connections is represented in Figure 1B. Three simulation conditions were created: baseline with a normal aortic valve, moderate aortic stenosis, and severe aortic stenosis. AS was simulated by increasing the aortic valve resistance parameter, confirmed by presence of the desired mean systolic pressure gradient between the LV and the ascending aorta. During the portion of systole when LV pressure exceeded aortic pressure, the difference between the two was calculated at each simulation timestep, and the average was taken to determine the mean gradient across the aortic valve. The baseline aortic valve resistance parameter was 1.0e-9 MPa^∗^s/mm^3^ with a mean gradient of <2 mmHg. Moderate aortic stenosis was achieved with resistance of 5.0e-9 MPa^∗^s/mm^3^ producing gradient of 20 mmHg, and severe aortic stenosis was created with 1.0e-8 MPa^∗^s/mm^3^ with gradient of 40 mmHg.

The unloaded heart was initialized in a zero-stress state obtained from iterative methods described by Sellier (2011) and Rausch et al. (2017), based on loaded *in vivo* images. At the start of the simulation, pressures within each cavity were ramped from zero to physiologic values at 70% of the diastole phase: right atrium 2 mmHg, right ventricle 2 mmHg, pulmonary artery 8 mmHg, left atrium 4 mmHg, left ventricle 4 mmHg, aorta 80 mmHg, systemic arterial chamber 80 mmHg, systemic venous chamber 2 mmHg. Results for analysis were obtained from the third cardiac cycle. Additional resistance and compliance parameters in the circuit were defined: systemic arterial resistance 1.4e+02 MPa^∗^s/mm^3^, systemic venous resistance 9.7e-1 MPa^∗^s/mm^3^, tricuspid valve resistance 2.5e-1 MPa^∗^s/mm^3^, pulmonary valve resistance 9.7e-1 MPa^∗^s/mm^3^, pulmonary vascular resistance 1.1e+1 MPa^∗^s/mm^3^, the mitral valve resistance 2.3e+0 MPa^∗^s/mm^3^, pulmonary compliance 7.5e+6 mm^3^/MPa, systemic venous compliance 4.5e+7 mm^3^/MPa, and systemic arterial compliance 2.25e+6 mm^3^/MPa.

Cardiac cycles were simulated with Abaqus FEA (Simulia^TM^, Johnston, Rhode Island, United States) with an LV ejection fraction (LVEF) of 60% for all three simulation conditions, with end-diastolic volume of 136–138 ml. The same LV geometry and mass were used across all three simulations for control and represented a non-remodeled heart. A complete cardiac cycle occurred over 0.7 s. The heart model is constrained in space by fixed node sets at the cut planes of the aortic arch, pulmonary trunk, and superior vena cava. An acceptable steady state was achieved after running three consecutive cardiac cycles, with further cycles producing <5% variation in the model’s chamber pressures. LV myofiber stress and strain values were obtained at end-diastole and peak systole (defined as the point at which greatest LV pressure was generated). Data are expressed as mean ± standard deviation. *T*-tests were used for statistical comparison of continuous variables.

## Results

LV pressure-volume loops and LV-aorta pressure-time curves for the different degrees of AS are shown in Figures 1C,D. Aortic systolic pressure was 112–114 mmHg while diastolic pressure was 56–57 mmHg, representing a normal human physiologic range. A mean systolic gradient of 20 mmHg between the LV and aorta represented moderate AS. Conditions producing a mean systolic gradient of 40 mmHg represented severe AS, and clinically would serve as an indication for aortic valve replacement (Nishimura et al., 2014; Baumgartner et al., 2017).

**FIGURE 2:**
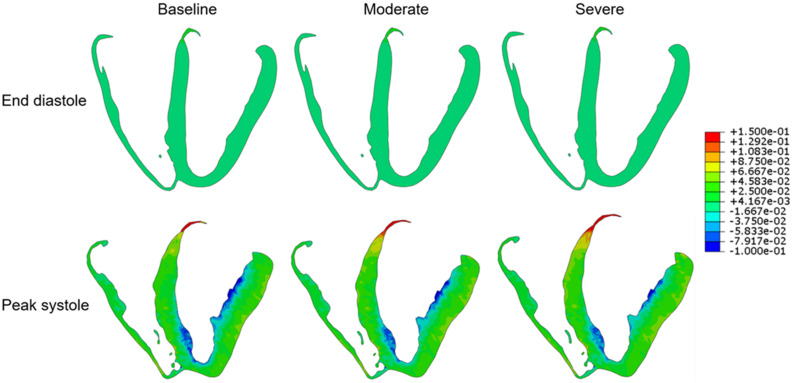
Long-axis cross-sectional views of the left ventricle demonstrating myofiber stress at end diastole (upper row) and peak systole (lower row) for each of the three simulation conditions: baseline, moderate aortic stenosis, and severe aortic stenosis.

Global mean LV myofiber stress and strain values at end-diastole and peak systole for each simulation condition are listed in Table 1. Long-axis LV cross-sectional myofiber distribution at end-diastole and peak systole are seen in Figure 2. The endocardial regions harbor predominantly negative myofiber stress at peak systole, a product of LV contraction physiology that has been observed in other LV models (Genet et al., 2014; Sack et al., 2016). Peak systolic myofiber stress increased progressively with increasing degree of AS, whereas end-diastolic stress across all conditions varied minimally with values <1.0 kPa. The mean global LV myofiber stress was significantly different between moderate AS and baseline (*p* < 0.01) as well as between severe AS and baseline (*p* < 0.001).

**TABLE 1:** Global myofiber stress and strain in the left ventricle.

Simulation condition	Myofiber stress		Myofiber strain	
	End-diastole (kPa)	Peak systole (kPa)	End-diastole (%)	Peak systole (%)
Baseline (no aortic stenosis)	0.31 ± 1.36	9.31 ± 10.33	+5.80 ± 4.74	−22.40 ± 8.73
Moderate aortic stenosis	0.32 ± 1.39	13.13 ± 10.29	+5.94 ± 4.82	−22.24 ± 8.91
Severe aortic stenosis	0.35 ± 1.44	16.18 ± 10.59	+6.16 ± 4.90	−21.97 ± 9.18

**FIGURE 3:**
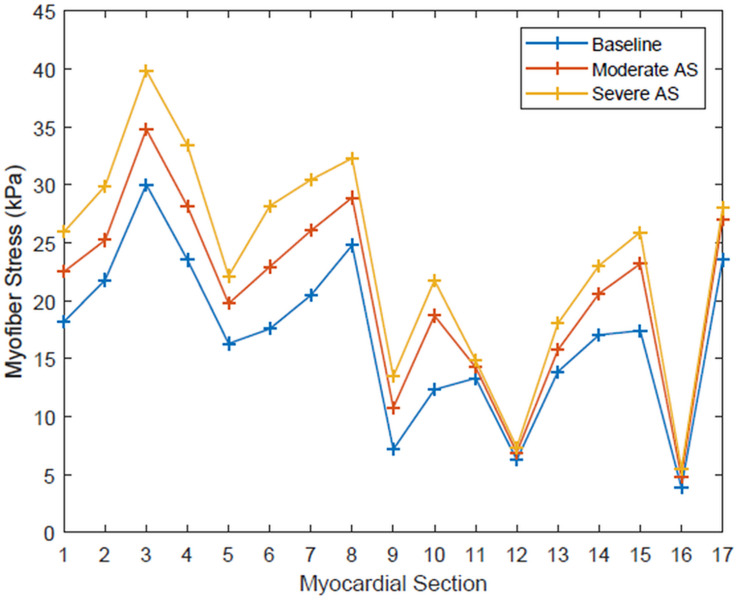
Systolic myofiber stress for each of the 17 standardized myocardial regions for each simulation condition: baseline, moderate aortic stenosis, and severe aortic stenosis.

Regional segmentation of the LV was performed in accordance to the American Heart Association standardized myocardial regions, creating 17 segments of LV myocardium based on anatomic location and coronary perfusion territories (Cerqueira et al., 2002). The mean myofiber stress of each of these segments is shown in Figure 3. The range of peak systolic stress was 3.89 to 30.03 kPa for the baseline simulation, 4.81 to 34.79 kPa for moderate AS, and 5.50 to 39.87 kPa for severe AS. Segments that had the greatest peak myofiber stress from the basal, mid, and apical regions were segment 3 (basal inferoseptal), segment 8 (mid-anteroseptal), and segment 17 (apex), and this finding was consistent across all three simulation conditions.

## Discussion

Aortic stenosis has typically been graded by hemodynamic parameters that can be measured on echocardiography: mean pressure gradient across the valve, effective orifice area, and peak blood jet velocity. However, a big limitation is that all of these are derived from other specific parameters and are flow-dependent. To better understand disease pathophysiology and find new clinically useful markers, we created a realistic human LV model with coupled circulatory system parameters, and isolated moderate and severe AS. This is one of the first computational studies to specifically investigate the impact of AS on LV myofiber stress. In this model, moderate stenosis provided peak LV myofiber stress 1.4 times greater than baseline, and severe stenosis yielded peak stress of 1.7 times greater than baseline. With all other parameters being held nearly equal (LVEF, aortic blood pressure, LV geometry), it is an anticipated physiologic response that higher degrees of afterload will increase the amount of stress experienced by the LV.

LV systolic performance and ventricular stress have been investigated as prognostic markers for AS patients (Wachtell, 2008). After all, chronic exposure to increased afterload triggers remodeling, leading to LV hypertrophy and eventual dysfunction, culminating in congestive heart failure. However, determining stress values from clinical imaging alone presents substantial limitations, such as not accounting for the myocardial material properties, or permitting prediction about how wall stress may change under different physiologic conditions or after treatment of AS. One study calculated end-systolic wall stress in 78 symptomatic and 91 asymptomatic patients and defined severe AS by aortic valve area ≤1 cm^2^ (Carter-Storch et al., 2019). End-systolic wall stress was estimated by measuring LV wall thickness from cardiac magnetic resonance images, and LV end-systolic pressure from echocardiogram-derived mean gradients. The results indicated that end-systolic wall stress was significantly greater in symptomatic patients at 9.6 kPa than in the asymptomatic patients at 7.6 kPa. Symptomatic patients also had markers of more severe AS and LV dysfunction, with lower LVEF and smaller indexed aortic valve areas. These results illustrate the potential clinical utility of LV stress as a marker of disease severity. Even with the aforementioned limitations, the authors found significant differences in the stresses, indicative of the critical role it has in the disease state. Differences between that study and ours in terms of patient population and methods of calculating wall stress make direct comparison of the numeric results challenging, but theirs are in range of our computationally derived myofiber stress results.

In a study of severe AS patients in which LV contractility was correlated with overall survival, end-systolic wall stress was greater in patients with LVEF <60% than in those with LVEF ≥60% (9.48 kPa vs. 7.91 kPa; *p* < 0.001) at the time severe AS was diagnosed (Ito et al., 2020). Furthermore, patients in the LVEF <60% group had significantly worse survival than those in the LVEF ≥60% group. Classification of the patient cohort by an end-systolic wall-stress threshold of 14.5 kPa, a value estimated two standard deviations above the mean in a population study by Aurigemma et al. (1994) also yielded significantly worse survival in patients with >14.5 kPa end-systolic wall stress. Six-year follow-up indicated cumulative survivals of 20% (>14.5 kPa group) vs. 45% (≤14.5 kPa group). This provides additional support for wall stress in the clinical evaluation and prognostication of patients with AS (Aurigemma et al., 1994; Ito et al., 2020).

The aforementioned studies were based on routine clinical data and imaging available, with wall stress calculation techniques based on Laplace’s Law. However, results derived from Laplace’s Law are hindered by assumptions of uniform chamber geometry, a relatively thin chamber thickness relative to radius, and do not account for the myocardial material property. Additionally, results derived using Laplace’s Law cannot provide detailed information on transmural distribution of wall stress nor local variations if focal ventricular pathology were to exist (Zhang et al., 2011).

The ability to create patient-specific finite element models would offer more accurate wall- stress results, as well as more insight into the ventricular pathophysiology. One of the earliest published finite element studies exploring the link between aortic valve pathology and LV stress was done on aortic insufficiency patients (Wollmuth et al., 2006). Patient-specific LV geometries were obtained from cardiac magnetic resonance imaging in patients with moderate-severe aortic insufficiency before and after aortic valve replacement, as well as in control volunteers without aortic valve disease. Maximum principal LV end systolic stress was significantly elevated in patients with aortic insufficiency before aortic valve replacement, relative to controls (10.6 vs. 9.12 kPa; *p* < 0.026). After aortic valve replacement, LV end systolic stress decreased to 7.08 kPa. These results help link the clinical benefit of aortic valve replacement with LV biomechanics data, but are limited by the use of finite element models with fairly few elements and assigning a linearly elastic, isotropic material model to the myocardium. Aside from the difference in the aortic valve disease studied by Wollmuth et al., our LV model uses a highly refined mesh for the finite element model, and a sophisticated myocardial material model that accounts for microstructure, active, and passive properties. The complexities of the myocardium need to be accounted for in the material law in order to obtain the most accurate results.

Analysis of regional myocardial biomechanics is facilitated with a solid, finite-element model. Our data from each of the 17 standardized myocardial segments reveals a range of stress at peak systole, with anatomic regions each harboring regions of localized peak stress. Examining how this ventricular stress profile may vary among patients, or shift as disease progresses, can offer a unique biomechanical profile for each patient. For example, Jung et al. classified cardiac computed tomography images of normal control patients and those with severe AS by physical parameters associated with the 17 segments (Jung et al., 2016). Two and three-dimensional parameters were found to discriminate between the two patient groups, with the severe AS group having greater LV wall thickness, segment mass, and surface area. Among the segments, a range of values exists for each parameter being evaluated. For instance, segment thickness in AS patients ranged from 11.4 to 16.4 mm, whereas area ranged 8.3–13.7 cm^2^. Although differences in ventricular topography and dimensions between the two groups are not surprising, the study illustrates the added benefit of using regional segments to perform more in-depth analysis of the LV.

In another study, the 17 segments were used to map the distribution of myocardial fibrosis in AS patients by analyzing gadolinium-contrast cardiac magnetic resonance imaging (Weidemann et al., 2009). Patients with more severe disease had fibrosis predominantly localized to the basal segments. Although the impact of myocardial fibrosis on LV myofiber stress remains a topic for further study, regional segmentation offers better understanding of the pathologic derangements occurring within the ventricle that are not necessarily homogenously distributed. Global LV markers of performance in conjunction with regional analysis can offer the most in-depth understanding of LV derangements.

With advances in computational modeling techniques, cardiovascular imaging, and a detailed understanding of myocardial material properties, patient-specific models of clinical utility are within reach and poised for more translational research roles. Non-invasive means of determining parameters to tune patient-specific models exist or are being perfected (Gray and Pathmanathan, 2018; Dabiri et al., 2019; Mineroff et al., 2019; Namasivayam et al., 2020). Enhanced constitutive myocardial material models have been developed to account for presence of fibrosis (Wang et al., 2016; Hasaballa et al., 2019). Ways to integrate this data into clinical practice will first require larger scale studies that have many patient-specific models. Clinical and computational data needs to be correlated with patient outcome, for example, whether the patient ultimately required aortic valve replacement and how AS impacted LV function over time. This correlation will help with the clinical validation of computationally derived data and establish its place in clinical decision-making algorithms.

### Limitations

The primary limitation of this study is that our model represents an idealized human heart geometry and simulates AS without any LV remodeling. The material properties were based on established, comprehensive models that describe normal myocardial physiology, whereas AS is often a progressive condition accompanied by a degree of LV remodeling and fibrosis. Future studies with computational modeling should incorporate patient-specific ventricular geometries, myocardial material properties, and should address all possible cardiovascular derangements in presence of AS.

## Conclusion

In this study, we used a realistic human heart model with coupled circulatory system to simulate AS and quantify the LV myofiber stresses. This preliminary investigation used computational methods to better assess the role of LV-aortic coupling in the pathophysiology of aortic stenosis. Our goal is to apply computationally derived data toward patient-specific assessment of AS to guide management and intervention before irreversible LV remodeling occurs.

## Data Availability Statement

The raw data supporting the conclusions of this article will be made available by the authors, without undue reservation, to any qualified researcher.

## Author Contributions

AW, YW, SP, and JG were involved in the conception, design of the study, and analysis of the results. YW ran simulations and provided data for analysis. JY and KS offered guidance on the conditions for the computational simulations and assisted in conducting the data analysis. AW, AH, TD, and JG were involved in data analysis, interpretation of results, and the clinical concepts of this study. All authors contributed to the article and approved the submitted version.

## Conflict of Interest

YW was employed by the company Thornton Tomassetti Lifesciences Division. KS is currently employed by the company Medtronic Inc. JY was employed by the company Dassault Systemes Simulia Corp. The remaining authors declare that the research was conducted in the absence of any commercial or financial relationships that could be construed as a potential conflict of interest. The reviewer HG declared a past co-authorship with one of the authors JY to the handling editor.
